# Efficacy and Tolerability of Second and Third Generation Anti-epileptic Drugs in Refractory Epilepsy: A Network Meta-Analysis

**DOI:** 10.1038/s41598-017-02525-2

**Published:** 2017-05-31

**Authors:** Chuanjun Zhuo, Ronghuan Jiang, Gongying Li, Mingjing Shao, Ce Chen, Guangdong Chen, Hongjun Tian, Jie Li, Rong Xue, Deguo Jiang

**Affiliations:** 1 Department of Psychiatry, Wenzhou Seventh People’s Hospital, Wenzhou Zhejiang, 325005 China; 2 Department of Psychiatry, Tianjin Anding Hospital, Tianjin, 300222 China; 3 Institute of Mental Health, Jining Medical University, Jining, Shandong 272067 China; 4 Department of Psychiatry, Tianjin Anning Hospital, Tianjin, 300222 China; 5 Department of Psychological Medicine, Chinese PLA (People’s Liberation Army) General Hospital, Chinese PLA (People’s Liberation Army) Medical School, Beijing, 100853 China; 6 Department of Cardiology, China-Japan Friendship Hospital, Beijing, 100029 China; 7 Department of Neurology, Tianjin Medical University General Hospital, Tianjin, 300052 China

## Abstract

This study was proposed to compare the relative efficacy and tolerability of the second and third generation AEDs for refractory epilepsy. The 50% responder rate (RR) was selected as the efficacy outcome whereas the incidence of dizziness and somnolence were considered to evaluate the tolerability of AEDs. Odds ratio (OR) and their 95% credible interval (*CrI*) were obtained using a consistency model and surface under the cumulative ranking curve (SUCRA) value was calculated to rank AEDs. Topiramate appeared to be significantly more effective than placebo, eslicarbazepine acetate, perampanel, pregabalin, zonisamide, gabapentin and lamotrigine with respect to the 50% RR (all OR > 1). Patients who were managed by eslicarbazepine acetate, perampanel, oxcarbazepine, topiramate and pregabalin were more likely to suffer from dizziness compared to those who receive placebo (all OR > 1). Perampanel, topiramate and pregabalin were related to elevated risks of somnolence compared to placebo (all OR > 1). Moreover, topiramate ranked highest with respect to 50% RR (SUCRA = 0.968) whereas levetiracetam appeared to have balanced efficacy and tolerability (SUCRA = 0.769, 0.743, 0.604 and 0.659). In conclusion, topiramate was the most efficacious AED, while levetiracetam was able to provide patients with balanced efficacy and tolerability.

## Introduction

Drug-resistant epilepsy (DRE), also called medically intractable epilepsy was defined as “a failure of adequate trials of two tolerated, appropriately chosen and used anticonvulsant drug schedules (whether as monotherapies or in combination) to achieve sustained seizure freedom” according to the International League Against Epilepsy (ILAE)^1^ Approximately 1 million people in the United States continue suffering from seizures despite adequate treatment with anti-epileptic drugs (AEDs), accounting for 40% of patient amount^2^. DRE can be associated with developmental delay in infants and young children, and severe disability and morbidity in older children and adults, as well as a mortality rate 5–10 times that of the general population^3^. There is a prevalent assumption declaring that refractory epilepsy is developed from the onset, however, this assumption may not be very convincible and a large number of patients develop intractable epilepsy despite that they response well to AEDs in the early stages^4^.

AEDs are appropriate for patients with following features: at least two epileptic seizures; abnormal results obtained from imaging, neurological exam or electroencephalography (EEG); family histories of seizures^5^. The primary objective of using AEDs for epilepsy is to control unexpected seizures while minimizing the corresponding side effects resulted from AEDs. Compared with other options such as surgical procedures, implantable devices and dietary therapies, AEDs have several advantages including excellent oral absorption and bioavailability. Moreover, the second and third generation AEDs are of equivalent efficacy and safer than the older ones and have several significant advantages including reduced drug-drug interactions, less life-threatening adverse events and less negative impact on cognitive functions^6^. Despite of improved efficacy and tolerability, the second and third generation AEDs are still far away from the ideal ones which are able to fully control seizures without significant impact on the life quality of patients while being highly affordable^7^.

The choice of an appropriate AED is a matter with huge complexity and it is unlikely to predict whether a patient will have a favorable response to an AED merely based on clinical features or laboratory results^8^. Several factors should be taken into consideration for clinical decision-making: monotherapy or multidrug therapy; the corresponding side effects; drug-drug or drug-food interactions; the corresponding costs and availability; the ability of patients to endure and manage side effects. Since the number of AEDs approved by the FDA has been increased dramatically in the past 15 years, evaluating the available AEDs with different mechanisms are essential in clinical practices. Besides, the development of new drugs is costly and risky mainly due to the fact that we have incomplete knowledge about the resistance to AEDs^9^. Therefore, undertaking a system review and evidence synthesis may help clinicians and drug developers understand and improve various mechanisms of AEDs as well as the corresponding adverse effects which are often underestimated by clinicians.

Network meta-analysis (NMA) is an objective way of comparing alternative treatments where direct treatment comparisons have not been made by comparative effectiveness researches. In our review, we conducted a NMA of randomized control trials (RCTs) to evaluate twelve AEDs including eslicarbazepine acetate, levetiracetam, retigabine, tiagabine, vigabatrin, perampanel, oxcarbazepine, lamotrigine, topiramate, pregabalin, zonisamide and gabapentin with respect to their efficacy and tolerability for refractory epilepsy. Our paper aims to provide an up-to-date and comprehensive synthesis of direct and indirect evidence to guide clinical application of AEDs for DRE.

## Material and Methods

### Literature search

Firstly, we carried out a systematic review for our research topic by reviewing the following elements in the current literature: population, interventions, outcomes, inclusion criteria, data extraction, quality assessment and data analysis. Then, a thorough literature search was carried out in multiple sources including PubMed, Embase and Cochrane Library from inception to 11 March 2016. The entire literature search process was conducted by two independent reviewers and the corresponding results were reviewed by a third reviewer. A well-designed search strategy comprising of multiple keywords with respect to the above AEDs and their marketing names were input into online databases in order to retrieve relevant articles. Apart from that, we manually searched the corresponding reference lists of relevant articles and such an extensive search may increase precision and minimize the risk of small study effect, publication bias as well as selective reporting.

### Inclusion criteria

The inclusion criteria were as follows: 1) the trial was RCT conducted on human subjects 2) the trial were focused on at least one AED including eslicarbazepine acetate, levetiracetam, retigabine, tiagabine, vigabatrin, perampanel, oxcarbazepine, lamotrigine, topiramate, pregabalin, zonisamide and gabapentin; 3) the diagnosis of epilepsy (partial seizure) was confirmed by brain CT scan, EEG, or MRI; 4) at least one efficacy or tolerability endpoint was assessed in the trial; 5) data can be extracted from studies to implement NMA. The primary outcome in our study is the 50% responder rate (RR) which is defined as the percentage of patients in the sample whose long term frequency of seizures was reduced by 50%. Endpoints with respect to tolerability included dizziness and somnolence which are commonly observed among patients. Screening of literatures was conducted by carefully reviewing their titles, abstracts as well as matching their contents with the corresponding selection criteria. Ineligible studies or duplicated studies were removed from the list prior to data extraction. Observational studies were also excluded from the eligible list.

### Data extraction and synthesis

Each variable extracted from eligible studies was clearly defined earlier in order to accomplish our research purpose. A data extraction spreadsheet was used to extract relevant data from individual studies and this process was implemented by two independent reviewers. Data extraction results were compared and any disagreement was resolved by discussion. Missing data contained in the individual studies were not imputed in the data extraction spreadsheet. Data with respect to the same AED within the same study may be combined if they only differ in doses.

### Statistical data analysis

Pairwise comparison between AEDs and placebo was visualized by forest plots and summary statistics such as odds ratios (ORs) together with their 95% confidence intervals (CIs) were used to assess the relative efficacy and tolerability of AEDs. Heterogeneity across individual studies was assessed by using the Cochran Q (Chi-squared) and Higgins I-squared statistics which quantified the percent of total variation due to between study heterogeneity^10^. Moreover, the implementation of NMA is based on the Bayesian Framework and non-informative prior probabilities were used in the Bayesian statistical approach. All statistical analysis and graphical procedures were conducted by R software (Version 3.2.4, The R Project for Statistical Computing) in conjunction with the GeMTC package. The random effect assumption was adopted in our NMA and the consistency model was generated from the GeMTC package. The competing AEDs were ranked based on their corresponding surface under the cumulative ranking curve (SUCRA) which indicates the cumulative probability that an AED is among the top n treatments and a cumulative ranking plots was created from R software in order to compliment the numerical summary of SUCRA. If there is clear evidence of inconsistency between direct evidence and indirect with respect to a specific comparison, then the consistency model was replaced by the inconsistency model in the GeMTC package.

## Results

### Basic characteristics of included studies and patients

**Figure 1:**
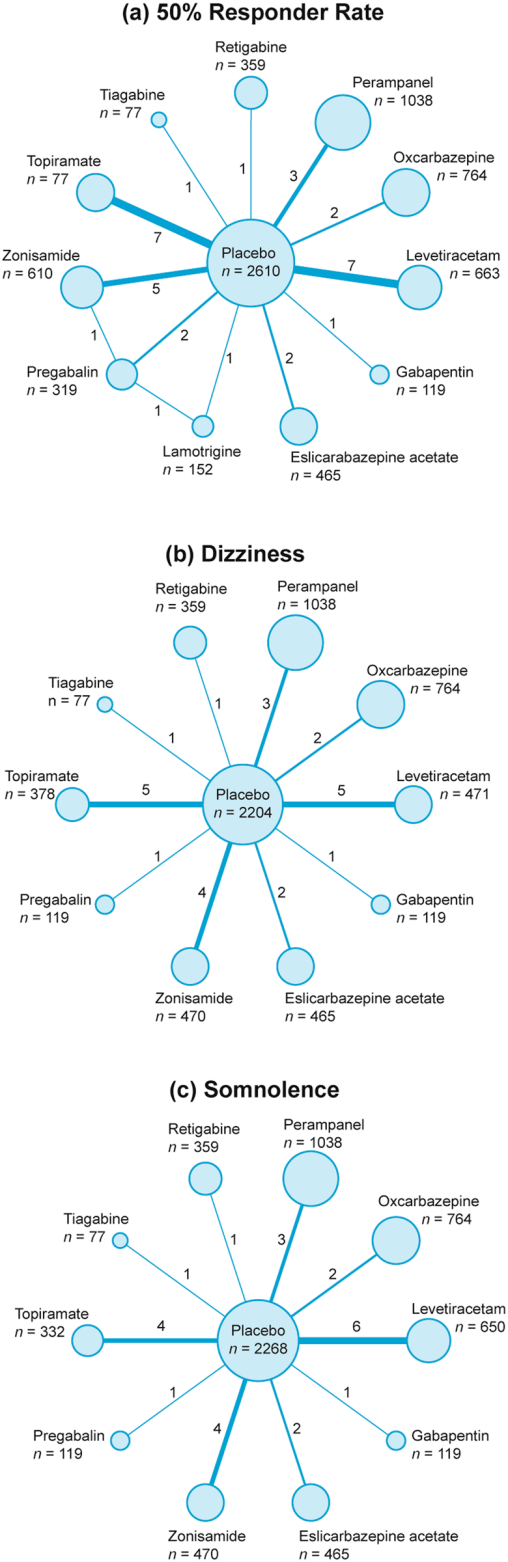
Network diagram for 50% responder rate, dizziness and somnolence.

According to the prior inclusion criteria, a total of 32 studies involving 7,658 patients were included in our NMA^11–42^. Details of flow chart were shown in Figure S1. Of the 32 trials, 30 were placebo-controlled, one was active-controlled (Zonisamide vs. Pregabalin) and one was a three-arm study (Pregabalin vs. Lamotrigine vs. Placebo). Detailed characteristics of included studies such as authors, publication year, mean age, AEDs, dose, efficacy or tolerability endpoint are displayed in Table 1. The detailed direct and indirect comparison with respect to each endpoint was illustrated by the network plot (Fig. 1) in which the node size was proportional to the number of subjects involved in each AED and the thickness of connected lines between two interventions was proportional to the number of comparisons between these two interventions. Patients included in this NMA all suffered from drug-resistant epilepsy, together with previous AED medication history. Besides, the seizure types were complicated, including simple partial, complex partial, secondarily generalized.

**Table 1:** Basic characteristics of included clinical trials.

Reference	Mean age	Intervention (N)	Dose^*^	50%RR	Treatment adverse events	
					Dizziness	Somnolence
Faught, E.^21^	36.9	Eslicarbazepine acetate(136); Placebo(45)	200–600 mg/d	Yes	Yes	Yes
Gil-Nagel, A.^25^	36.4; 37.7	Eslicarbazepine acetate(165); Placebo(87)	800, 1200 mg/d	Yes	Yes	Yes
French, J. A.^24^	36.1; 34.4	Eslicarbazepine acetate(250); Placebo(136)	8, 12 mg/d	Yes	Yes	Yes
French, J. A.^23^	36.3; 35.6	Eslicarbazepine acetate(267); Placebo(121)	8, 12 mg/d	Yes	Yes	Yes
Elger, C.^19^	39.1; 37.0	Eslicarbazepine acetate(300); Placebo(102)	400–1200 mg/d	Yes	Yes	Yes
Appleton, R.^11^	8.5; 8.4	Gabapentin(119); Placebo(128)	600–1800 mg/d	Yes	Yes	Yes
Wu, X. Y.^38^	32.7; 32.8	Levetiracetam(102); Placebo(100)	1000–3000 mg/d	Yes	Yes	Yes
Zhou, B.^42^	28.2; 31.3	Levetiracetam(13); Placebo(11)	750 mg/d	Yes		
Ben-Menachem, E.^15^	37.0; 36.0	Levetiracetam(181); Placebo(105)	3000 mg/d	Yes		Yes
Shorvon, S. D.^34^	37.0	Levetiracetam(212); Placebo(112)	1000, 2000 mg/d	Yes	Yes	Yes
Xiao, Z.^39^	32.8; 32.5	Levetiracetam(28); Placebo(28)	3000 mg/d	Yes	Yes	Yes
Tsai, J. J.^37^	32.8; 31.7	Levetiracetam(47); Placebo(47)	1000–2000 mg/d	Yes	Yes	Yes
Betts, T.^16^	37.5; 35	Levetiracetam(80); Placebo(39)	6000 mg/d	Yes	Yes	Yes
French, J. A.^22^	38.8; 39.1	Oxcarbazepine(245); Placebo(121)	1200, 2400 mg/d	Yes	Yes	Yes
Barcs, G.^12^	34.5, 34.3	Oxcarbazepine(519); Placebo(173)	600, 1200, 2400 mg/d	Yes	Yes	Yes
Krauss, G. L.^27^	34.0; 33.4	Perampanel(521); Placebo(185)	2, 4, 8 mg/d	Yes	Yes	Yes
Lee, B. I.^28^	33.3; 35.1	Pregabalin(119); Placebo(59)	150–600 mg/d	Yes	Yes	Yes
Baulac, M.^13^	39.8; 39.4; 39.1	Pregabalin(140); Lamotrigine(152); Placebo(141)	300, 600 mg/d; 300, 400 mg/d	Yes		
Brodie, M. J.^19^	37.6; 37.7	Retigabine(359); Placebo(179)	600, 900 mg/d	Yes	Yes	Yes
Kalviainen, R.^26^	36.4; 36	Tiagabine(77); Placebo(77)	12–30 mg/d	Yes	Yes	Yes
Privitera, M.^30^	35.5	Topiramate(143); Placebo(47)	600–1000 mg/d	Yes	Yes	Yes
Yen, D. J.^40^	31.4; 32.0	Topiramate(23); Placebo(23)	300 mg/d	Yes	Yes	
Sharief, M.^33^	35.4; 32.6	Topiramate(23); Placebo(24)	400 mg/d	Yes		Yes
Tassinari, C. A.^36^	32.9	Topiramate(30); Placebo(30)	600 mg/d	Yes	Yes	Yes
Zhang, L.^41^	72.6; 73.9	Topiramate(46); Placebo(40)	200 mg/d	Yes	Yes	
Ben-Menachem, E.^15^	34.1; 32.0	Topiramate(81); Placebo(52)	400–800 mg/d	Yes		
Faught, E.^20^	35.8; 34.2	Zonisamide(118); Placebo(85)	100, 200, 400 mg/d	Yes	Yes	Yes
Brodie, M. J.^18^	35.3; 36.5	Zonisamide(229); Placebo(120)	100–500 mg/d	Yes	Yes	Yes
Lu, Y.^29^	36.83; 29.81	Zonisamide(53); Placebo(51)	300–400 mg/d	Yes	Yes^**^	Yes^**^
Taghdiri, M. M.^35^	6.2; 5.9	Zonisamide(61); Pregabalin(60)	2–12 mg/kg/d; 5–15 mg/kg/d	Yes		
Schmidt, D.^32^	36.2; 33.4	Zonisamide(71); Placebo(68)	400–1200 mg/d	Yes	Yes	Yes
Sackellares, J. C.^31^	35.6; 36.4	Zonisamide(78); Placebo(74)	400–600 mg/d	Yes		

*Dose for placebo not specified; ^**^Numbers of patients are 52 and 50 for Zonisamide and placebo respectively.

### Results of NMA with respect to efficacy

**Figure 2:**
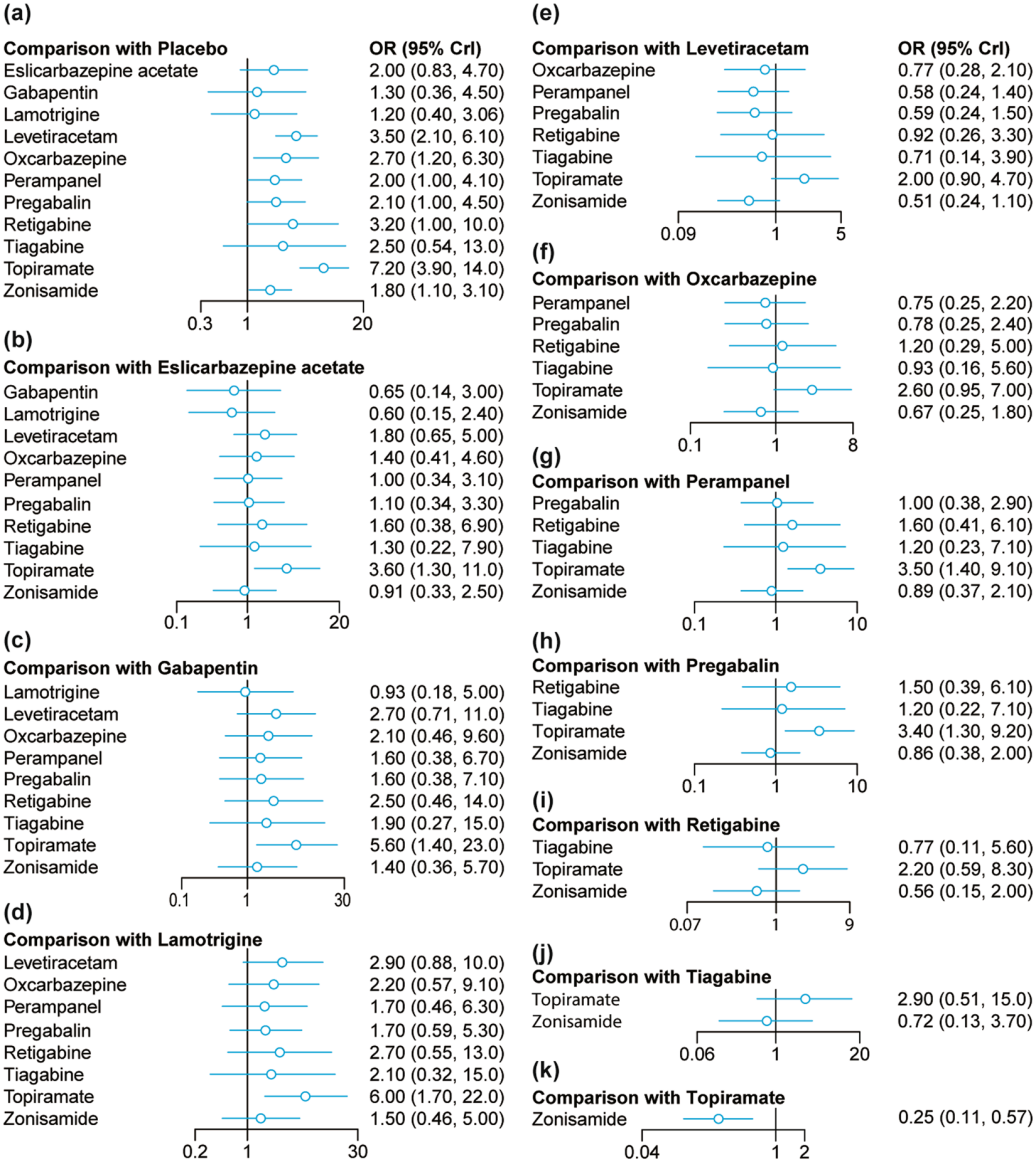
Forest plots for 50% responder rate.

The relative efficacy of AEDs was evaluated by using the 50% RR which was defined as the proportion of patients whose long-term seizures had been reduced by at least 50%. As suggested by Table 2, several AEDs demonstrated their significant effectiveness in terms of reducing the incidence of long-term seizures: topiramate, perampanel, oxcarbazepine, levetiracetam, pregabalin and zonisamide compared with placebo (all OR > 1). Furthermore, topiramate (OR = 7.09, 95% *CrI* = 3.93–13.36) appeared to be far more effective than several AEDs including eslicarbazepine acetate, perampanel, pregabalin, zonisamide, gabapentin and lamotrigine (all OR > 1). Therefore, we suspected that topiramate might exhibit the highest efficacy compared to other AEDs with respect to seizure reduction (Table 2, Fig. 2).

**Table 2:** NMA analysis results for 50% responder rate, dizziness and somnolence.

		Placebo	Esl. acetate	Levetiracetam	Retigabine	Tiagabine	Perampanel	Oxcarbazepine	Topiramate	Pregabalin	Zonisamide	Gabapentin	Lamotrigine
**50% RR**	**Placebo**		1.99 (0.83, 4.92)	**3.53 (2.12, 6.03)**	3.25 (0.99, 10.63)	2.50 (0.54, 12.94)	**2.02 (1.01, 4.09)**	**2.70 (1.16, 6.24)**	**7.09 (3.93, 13.36)**	**2.11 (1.01, 4.51)**	**1.81 (1.06, 3.16)**	1.26 (0.37, 4.45)	1.21 (0.40, 3.71)
	**Eslicarbazepine acetate**	0.50 (0.20, 1.20)		1.77 (0.64, 4.96)	1.64 (0.37, 6.87)	1.26 (0.21, 7.88)	1.02 (0.33, 3.12)	1.36 (0.40, 4.59)	**3.56 (1.23, 10.57)**	1.05 (0.34, 3.30)	0.91 (0.33, 2.49)	0.64 (0.14, 2.86)	0.61 (0.14, 2.48)
	**Levetiracetam**	**0.28 (0.17, 0.47)**	0.57 (0.20, 1.55)		0.92 (0.25, 3.32)	0.72 (0.14, 3.88)	0.57 (0.24, 1.36)	0.77 (0.29, 2.03)	2.02 (0.89, 4.58)	0.60 (0.24, 1.49)	0.52 (0.24, 1.10)	0.36 (0.09, 1.36)	0.34 (0.10, 1.19)
	**Retigabine**	0.31 (0.09, 1.01)	0.61 (0.15, 2.74)	1.09 (0.30, 3.99)		0.79 (0.11, 5.89)	0.63 (0.16, 2.46)	0.84 (0.20, 3.45)	2.20 (0.60, 8.36)	0.65 (0.16, 2.64)	0.56 (0.15, 2.12)	0.39 (0.07, 2.21)	0.37 (0.07, 1.90)
	**Tiagabine**	0.40 (0.08, 1.85)	0.79 (0.13, 4.67)	1.39 (0.26, 7.12)	1.27 (0.17, 9.02)		0.80 (0.14, 4.31)	1.06 (0.18, 6.13)	2.83 (0.50, 14.79)	0.83 (0.14, 4.63)	0.72 (0.13, 3.74)	0.50 (0.06, 3.70)	0.47 (0.07, 3.22)
	**Perampanel**	**0.49 (0.24, 0.99)**	0.98 (0.32, 3.01)	1.75 (0.74, 4.24)	1.60 (0.41, 6.37)	1.24 (0.23, 7.31)		1.34 (0.45, 3.87)	**3.50 (1.41, 9.08)**	1.03 (0.37, 2.95)	0.89 (0.37, 2.18)	0.63 (0.15, 2.58)	0.60 (0.16, 2.25)
	**Oxcarbazepine**	**0.37 (0.16, 0.87)**	0.74 (0.22, 2.50)	1.30 (0.49, 3.51)	1.20 (0.29, 4.93)	0.94 (0.16, 5.71)	0.75 (0.26, 2.23)		2.63 (0.95, 7.49)	0.78 (0.26, 2.47)	0.67 (0.25, 1.83)	0.47 (0.11, 2.08)	0.45 (0.11, 1.83)
	**Topiramate**	**0.14 (0.07, 0.25)**	**0.28 (0.09, 0.81)**	0.50 (0.22, 1.12)	0.46 (0.12, 1.67)	0.35 (0.07, 2.00)	**0.29 (0.11, 0.71)**	0.38 (0.13, 1.05)		**0.30 (0.11, 0.76)**	**0.26 (0.11, 0.59)**	**0.18 (0.04, 0.72)**	**0.17 (0.05, 0.60)**
	**Pregabalin**	**0.47 (0.22, 0.99)**	0.95 (0.30, 2.91)	1.68 (0.67, 4.21)	1.54 (0.38, 6.23)	1.20 (0.22, 7.03)	0.97 (0.34, 2.70)	1.29 (0.40, 3.85)	**3.35 (1.31, 9.04)**		0.86 (0.37, 1.97)	0.60 (0.14, 2.62)	0.57 (0.19, 1.77)
	**Zonisamide**	**0.55 (0.32, 0.94)**	1.10 (0.40, 3.05)	1.94 (0.91, 4.13)	1.78 (0.47, 6.61)	1.40 (0.27, 7.71)	1.12 (0.46, 2.74)	1.49 (0.55, 4.03)	**3.91 (1.70, 8.94)**	1.16 (0.51, 2.67)		0.70 (0.18, 2.75)	0.67 (0.20, 2.23)
	**Gabapentin**	0.79 (0.22, 2.72)	1.57 (0.35, 7.29)	2.78 (0.73, 11.00)	2.55 (0.45, 14.32)	1.99 (0.27, 15.75)	1.59 (0.39, 6.65)	2.12 (0.48, 9.44)	**5.57 (1.39, 22.96)**	1.66 (0.38, 7.17)	1.44 (0.36, 5.58)		0.94 (0.18, 5.28)
	**Lamotrigine**	0.82 (0.27, 2.53)	1.65 (0.40, 6.91)	2.91 (0.84, 10.10)	2.68 (0.53, 14.19)	2.12 (0.31, 14.75)	1.68 (0.44, 6.12)	2.24 (0.55, 8.87)	**5.82 (1.66, 21.52)**	1.74 (0.57, 5.31)	1.50 (0.45, 5.04)	1.06 (0.19, 5.64)	
		**Placebo**	**Esl. acetate**	**Levetiracetam**	**Retigabine**	**Tiagabine**	**Perampanel**	**Oxcarbazepine**	**Topiramate**	**Pregabalin**	**Zonisamide**	**Gabapentin**	
**Dizziness**	**Placebo**		**3.83 (1.21, 13.41)**	1.50 (0.68, 3.83)	4.06 (0.93, 18.37)	3.59 (0.72, 18.47)	**4.44 (1.93, 10.84)**	**2.99 (1.02, 8.62)**	**2.38 (1.07, 5.76)**	**6.41 (1.24, 38.10)**	1.55 (0.64, 3.84)	1.84 (0.17, 24.83)	
	**Eslicarbazepine acetate**	**0.26 (0.07, 0.82)**		0.40 (0.09, 1.75)	1.07 (0.15, 6.92)	0.92 (0.12, 7.15)	1.16 (0.25, 4.93)	0.79 (0.15, 3.72)	0.62 (0.14, 2.67)	1.67 (0.21, 13.95)	0.40 (0.09, 1.80)	0.48 (0.03, 7.98)	
	**Levetiracetam**	0.67 (0.26, 1.48)	2.52 (0.57, 11.34)		2.70 (0.46, 14.47)	2.37 (0.35, 14.52)	2.95 (0.82, 9.53)	1.99 (0.48, 7.47)	1.59 (0.45, 5.11)	4.29 (0.62, 28.74)	1.03 (0.29, 3.39)	1.22 (0.09, 19.26)	
	**Retigabine**	0.25 (0.05, 1.08)	0.94 (0.14, 6.65)	0.37 (0.07, 2.16)		0.89 (0.09, 8.03)	1.10 (0.20, 6.02)	0.74 (0.12, 4.56)	0.59 (0.11, 3.39)	1.59 (0.17, 14.56)	0.38 (0.06, 2.22)	0.45 (0.03, 8.96)	
	**Tiagabine**	0.28 (0.05, 1.40)	1.08 (0.14, 8.49)	0.42 (0.07, 2.82)	1.12 (0.12, 10.54)		1.24 (0.21, 7.78)	0.83 (0.12, 5.77)	0.66 (0.11, 4.15)	1.80 (0.17, 19.34)	0.44 (0.07, 2.74)	0.52 (0.03, 10.09)	
	**Perampanel**	**0.23 (0.09, 0.52)**	0.86 (0.20, 4.04)	0.34 (0.10, 1.22)	0.91 (0.17, 5.10)	0.81 (0.13, 4.80)		0.68 (0.16, 2.58)	0.54 (0.16, 1.81)	1.43 (0.22, 10.09)	0.35 (0.10, 1.20)	0.42 (0.03, 5.94)	
	**Oxcarbazepine**	**0.33 (0.12, 0.98)**	1.27 (0.27, 6.78)	0.50 (0.13, 2.08)	1.34 (0.22, 8.64)	1.21 (0.17, 8.52)	1.48 (0.39, 6.14)		0.80 (0.21, 3.14)	2.15 (0.31, 17.43)	0.52 (0.13, 2.08)	0.63 (0.04, 9.33)	
	**Topiramate**	**0.42 (0.17, 0.94)**	1.62 (0.37, 7.01)	0.63 (0.20, 2.24)	1.71 (0.29, 9.26)	1.52 (0.24, 9.29)	1.86 (0.55, 6.22)	1.25 (0.32, 4.82)		2.69 (0.41, 18.32)	0.65 (0.19, 2.23)	0.76 (0.06, 11.64)	
	**Pregabalin**	**0.16 (0.03, 0.81)**	0.60 (0.07, 4.78)	0.23 (0.03, 1.61)	0.63 (0.07, 5.95)	0.56 (0.05, 5.86)	0.70 (0.10, 4.52)	0.46 (0.06, 3.24)	0.37 (0.05, 2.45)		0.24 (0.03, 1.57)	0.29 (0.01, 6.88)	
	**Zonisamide**	0.64 (0.26, 1.56)	2.48 (0.56, 11.70)	0.97 (0.29, 3.50)	2.63 (0.45, 15.48)	2.29 (0.36, 14.88)	2.85 (0.83, 10.14)	1.91 (0.48, 7.71)	1.53 (0.45, 5.24)	4.09 (0.64, 30.12)		1.16 (0.09, 19.23)	
	**Gabapentin**	0.54 (0.04, 6.03)	2.10 (0.13, 31.45)	0.82 (0.05, 11.06)	2.23 (0.11, 39.58)	1.94 (0.10, 35.04)	2.41 (0.17, 31.79)	1.60 (0.11, 22.91)	1.32 (0.09, 16.93)	3.49 (0.15, 74.27)	0.86 (0.05, 10.62)		
**Somnolence**	**Placebo**		2.38 (0.81, 8.33)	1.63 (0.90, 3.09)	2.37 (0.63, 8.74)	0.90 (0.20, 3.86)	**2.40 (1.13, 5.48)**	2.15 (0.84, 5.36)	**2.86 (1.26, 6.54)**	**5.99 (1.12, 39.13)**	1.55 (0.70, 3.40)	2.02 (0.41, 9.99)	
	**Eslicarbazepine acetate**	0.42 (0.12, 1.23)		0.67 (0.17, 2.35)	1.00 (0.16, 5.13)	0.37 (0.05, 2.21)	1.01 (0.24, 3.90)	0.88 (0.18, 3.71)	1.18 (0.28, 4.73)	2.52 (0.31, 21.05)	0.65 (0.14, 2.46)	0.85 (0.10, 5.44)	
	**Levetiracetam**	0.61 (0.32, 1.12)	1.48 (0.43, 5.74)		1.44 (0.35, 6.21)	0.56 (0.11, 2.70)	1.48 (0.55, 4.05)	1.30 (0.41, 3.89)	1.74 (0.61, 4.82)	3.69 (0.61, 26.51)	0.95 (0.34, 2.55)	1.23 (0.23, 6.60)	
	**Retigabine**	0.42 (0.11, 1.59)	1.00 (0.19, 6.34)	0.70 (0.16, 2.87)		0.38 (0.05, 2.61)	1.01 (0.23, 4.75)	0.91 (0.18, 4.41)	1.20 (0.25, 5.68)	2.55 (0.30, 25.17)	0.65 (0.14, 2.96)	0.85 (0.11, 6.39)	
	**Tiagabine**	1.11 (0.26, 4.91)	2.68 (0.45, 18.55)	1.80 (0.37, 9.38)	2.64 (0.38, 19.08)		2.67 (0.51, 14.97)	2.37 (0.42, 13.52)	3.14 (0.58, 16.76)	6.65 (0.73, 70.06)	1.71 (0.33, 9.44)	2.25 (0.25, 19.36)	
	**Perampanel**	**0.42 (0.18, 0.89)**	0.99 (0.26, 4.15)	0.68 (0.25, 1.83)	0.99 (0.21, 4.30)	0.37 (0.07, 1.95)		0.89 (0.25, 2.89)	1.18 (0.36, 3.68)	2.47 (0.38, 19.09)	0.64 (0.21, 1.89)	0.84 (0.14, 4.69)	
	**Oxcarbazepine**	0.46 (0.19, 1.20)	1.13 (0.27, 5.68)	0.77 (0.26, 2.43)	1.10 (0.23, 5.67)	0.42 (0.07, 2.37)	1.13 (0.35, 3.98)		1.32 (0.39, 4.73)	2.83 (0.41, 22.82)	0.72 (0.22, 2.46)	0.96 (0.15, 5.85)	
	**Topiramate**	**0.35 (0.15, 0.80)**	0.85 (0.21, 3.60)	0.57 (0.21, 1.65)	0.83 (0.18, 3.97)	0.32 (0.06, 1.72)	0.85 (0.27, 2.76)	0.75 (0.21, 2.54)		2.09 (0.32, 16.51)	0.55 (0.17, 1.73)	0.71 (0.11, 4.22)	
	**Pregabalin**	**0.17 (0.03, 0.89)**	0.40 (0.05, 3.18)	0.27 (0.04, 1.64)	0.39 (0.04, 3.31)	0.15 (0.01, 1.37)	0.41 (0.05, 2.64)	0.35 (0.04, 2.41)	0.48 (0.06, 3.12)		0.25 (0.03, 1.66)	0.34 (0.03, 3.14)	
	**Zonisamide**	0.64 (0.29, 1.44)	1.54 (0.41, 6.91)	1.05 (0.39, 2.98)	1.54 (0.34, 7.09)	0.58 (0.11, 3.00)	1.56 (0.53, 4.87)	1.38 (0.41, 4.60)	1.82 (0.58, 5.91)	3.92 (0.60, 30.10)		1.32 (0.21, 7.50)	
	**Gabapentin**	0.49 (0.10, 2.42)	1.18 (0.18, 9.62)	0.81 (0.15, 4.43)	1.17 (0.16, 8.97)	0.44 (0.05, 4.01)	1.19 (0.21, 7.22)	1.04 (0.17, 6.75)	1.41 (0.24, 8.82)	2.98 (0.32, 36.22)	0.76 (0.13, 4.67)

### Results of NMA with respect to adverse events

**Figure 3:**
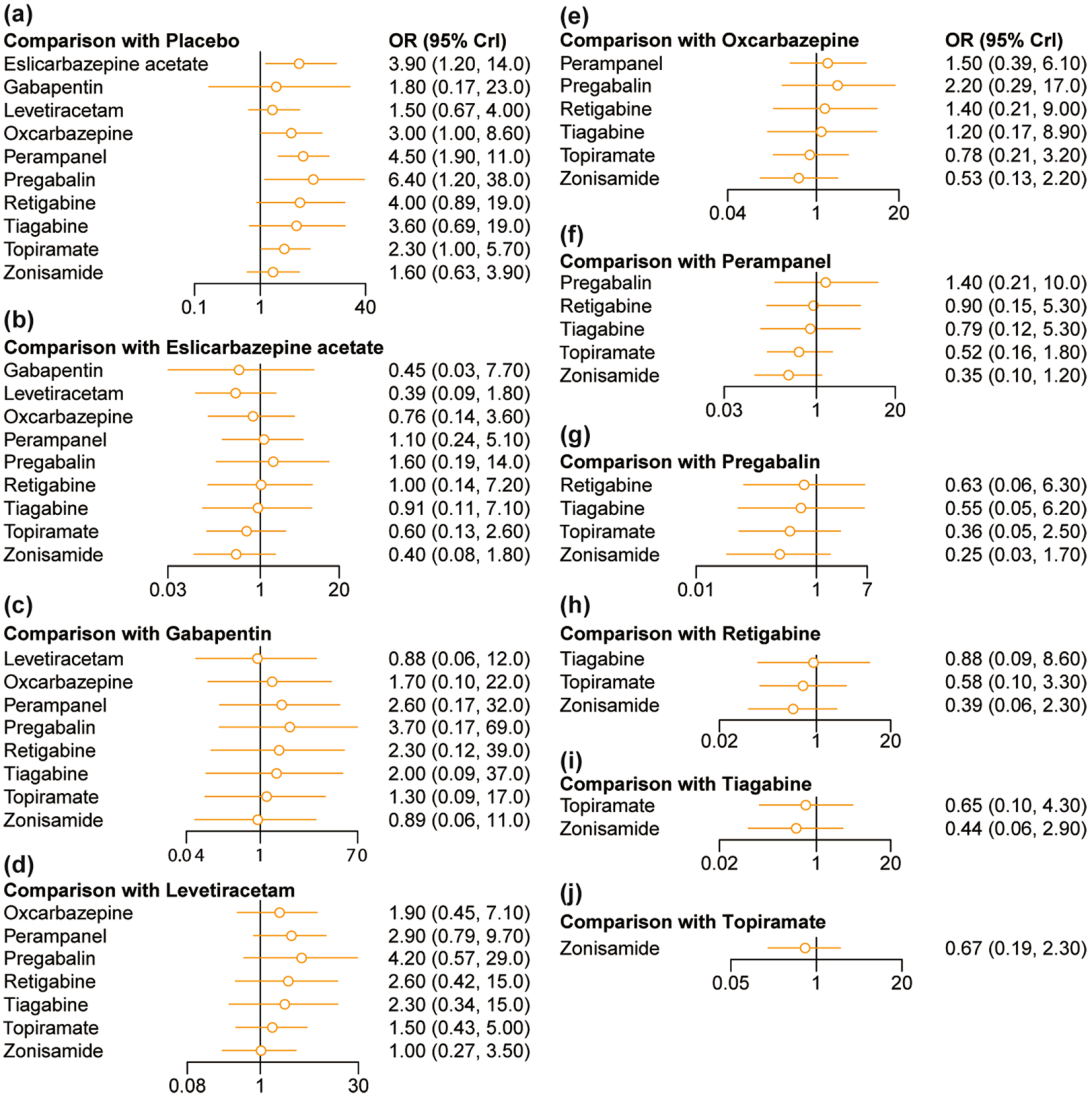
Forest plots for dizziness.

**Figure 4:**
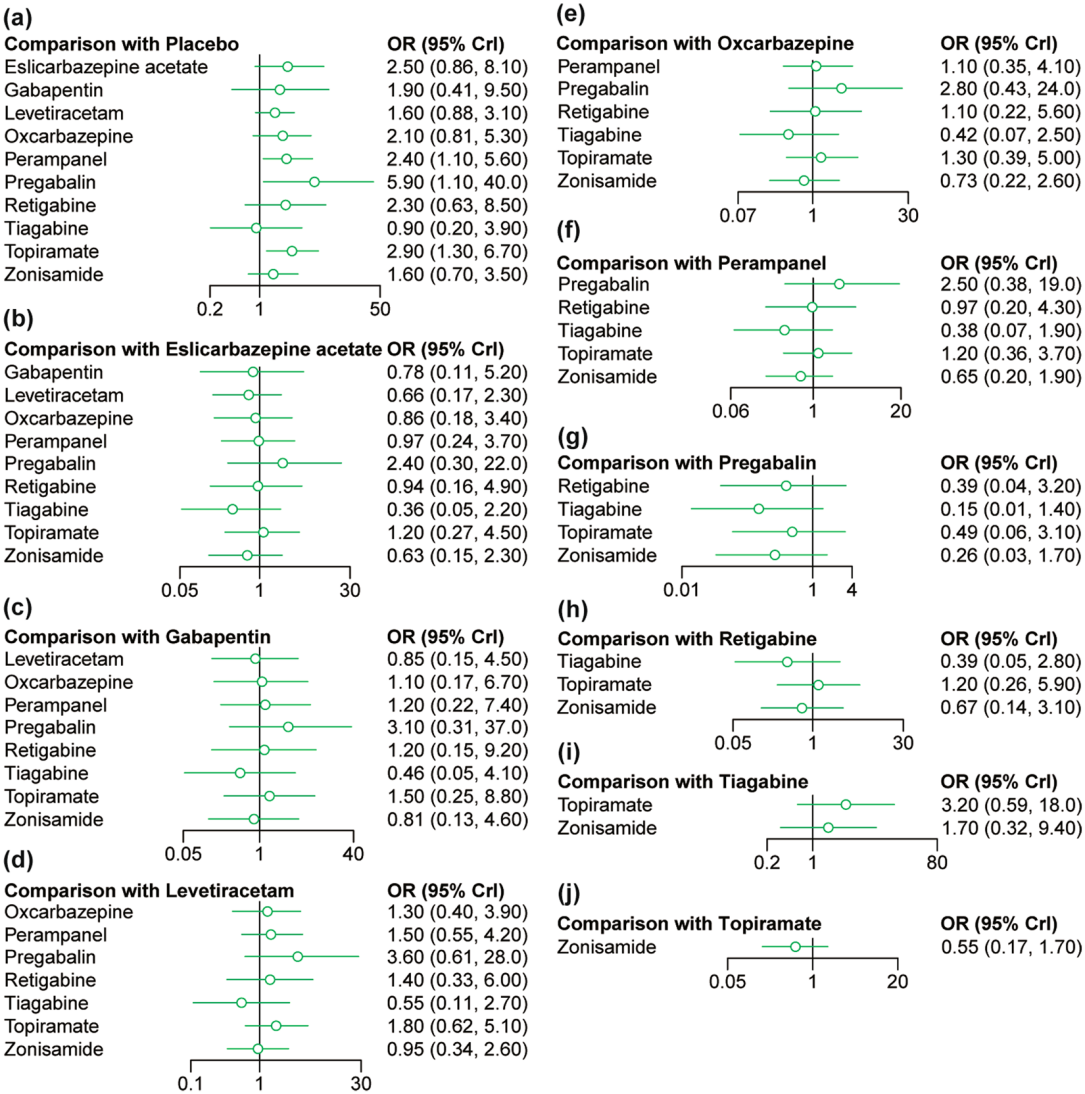
Forest plots for somnolence.

The incidence of three major adverse events including dizziness and somnolence were used to assess the tolerability of AEDs. As illustrated by Table 2 and Fig. 3, patients who were treated by eslicarbazepine acetate, perampanel, oxcarbazepine, topiramate and pregabalin were more likely to suffer from dizziness compared to those who receive placebo (all OR > 1). However, there was no significant difference in the risk of dizziness between any of the AEDs. Apart from that, patients who received perampanel, topiramate and pregabalin were at higher risks of suffering from somnolence compared to those who received placebo (all OR > 1; Table 2, Fig. 4).

### Ranks of AEDs by using SUCRA values

**Figure 5:**
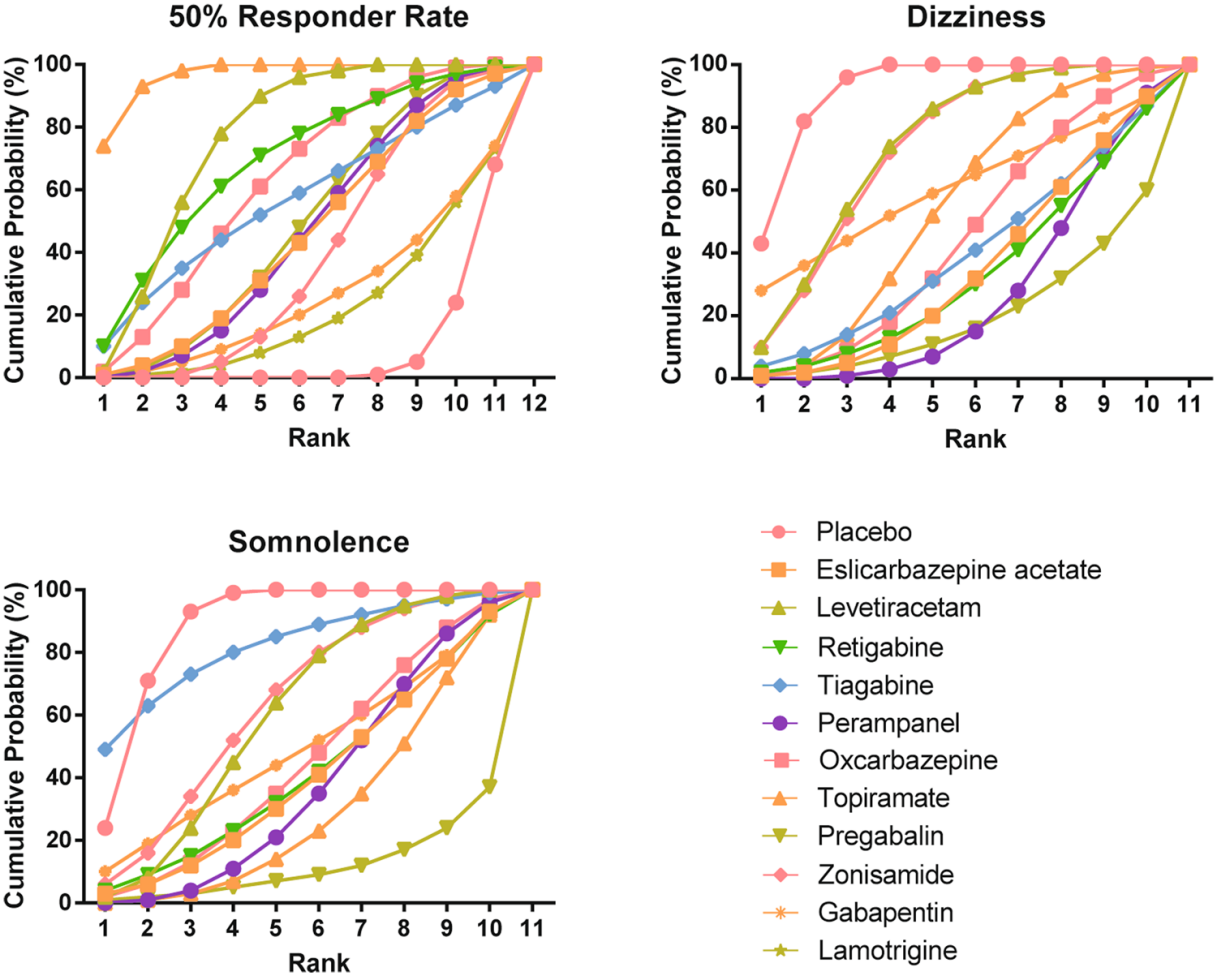
Cumulative probability diagram for 50% responder rate, dizziness, somnolence and headache.

One distinctive advantage of NMA based on the Bayesian Framework is its ability to rank the corresponding interventions by using their corresponding SUCRA values (Fig. 5). A higher SUCRA value in efficacy index provides evidence that it outperforms others, while in adverse effect a higher SUCRA value suggests a low probability of the occurrence of side effect. Topiramate (SUCRA = 0.968), levetiracetam (SUCRA = 0.769) and retigabine (SUCRA = 0.693) appeared to have the highest, second and third SUCRA values except for placebo with respect to 50% RR. However, lamotrigine appeared to be the least efficacious AED due to its lowest SUCRA value (SUCRA = 0.220) with respect to 50% RR. Also, levetiracetam (SUCRA = 0.743) and zonisamide (SUCRA = 0.735) appeared to be more tolerable than other AEDs with respect to the incidence of dizziness. Apart from that, tiagabine (SUCRA = 0.822) and zonisamide (SUCRA = 0.636) appeared to be more favorable than others with respect to somnolence. In general, topiramate appeared to be the most effective AED for managing seizures and levetiracetam had a balanced efficacy and tolerability in comparison to other AEDs since its corresponding SUCRA values of different endpoints all ranks relatively high (Table 3).

**Table 3:** SUCRA results for 50% responder rate, dizziness and somnolence.

Treatment	50%RR	Dizziness	Somnolence
Placebo	0.089	**0.921**	**0.887**
Eslicarbazepine acetate	0.458	0.344	0.401
Levetiracetam	**0.769**	**0.743**	0.604
Retigabine	**0.693**	0.328	0.413
Tiagabine	0.566	0.393	**0.822**
Perampanel	0.465	0.264	0.376
Oxcarbazepine	0.628	0.446	0.450
Topiramate	**0.968**	0.543	0.298
Pregabalin	0.489	0.199	0.117
Zonisamide	0.392	**0.735**	**0.636**
Gabapentin	0.261	0.605	0.489
Lamotrigine	0.220	—	—

## Discussion

Results from NMA indicated that topiramate might be more efficacious than other AEDs with respect to long-term seizures control. Meanwhile, DRE patients who were treated by levetiracetam, tiagabine or pregabalin were at lower risks of dizziness or somnolence. Apart from that, levetiracetam demonstrated the best combination of long-term efficacy and tolerability for DRE patients.

Topiramate is a new generation antiepileptic drug registered and introduced in 1995. It is usually applied in clinical practices as a monotherapy or polytherapy for managing DRE epilepsy^43^. Previous literature has indicated that topiramate has been widely used and seems to be far more effective than conventional anticonvulsants for its multiple effects on receptors and ion channels^44–46^. First of all, topiramate is able to block sodium-channel and may inhibit the process of synaptic conductance which is responsible for the transmission of epileptiform discharges^47^. Another key action of topiramate is its capability to impede excitatory glutamatergic transmission and thereby terminating seizure discharges^44^. Other actions of topiramate include the inhibition of calcium channels with high voltage activation as well as the inhibition of carbonic anhydrase activity that is linked with pH modulation^48, 49^. Furthermore, topiramate combined with conventional agents is likely to trigger an additional blockade of the sodium channel since it potentially interacts with other anticonvulsants to produce effects on protein binding^50^. Topiramate is characterized by linear pharmacokinetics, low protein binding and few active metabolites which result in fast absorption and oral bioavailability^46^. Preliminary data from long-term follow-up study also indicates that the efficacy of topiramate is more sustained in patients who experience localization-related epilepsy than in those who experience generalized epilepsy. All the above advantages of topiramate may explain its superiority over other AEDs with respect to the management of long-term seizures.

With respect to adverse effects, serious adverse effects associated with topiramate are rare but clinicians still have to be familiar with several mild to moderate adverse effects since these adverse effects particular for cognitive problems may result in the treatment discontinuation^51, 52^. Results from our NMA also suggested that epilepsy patients treated by topiramate were associated with significant increases in the risks of dizziness and somnolence compared to those who received placebo. However, cognitive adverse effects are not inevitable and tolerability of topiramate can be improved by setting low initial doses of 25 mg/day with slow titration^53, 54^.

Levetiracetam is another new AED for DRE. Our study has indicated that levetiracetam is the second most efficacious AED for control of long-term seizures and appears to have balanced efficacy and tolerability. A head-to-head comparison between levetiracetam and topiramate reveals that levetiracetam exhibits significantly higher retention rate and less side effects with equivalent efficacy compared with topiramate^55^. The above trend is strongly consistent with our NMA which indicated that topiramate was more efficacious than levetiracetam whereas the latter appeared to be far more tolerable with respect to the adverse events of dizziness and somnolence. Levetiracetam exhibits its unique antiepileptic mechanism by inhibiting high-voltage-activated Ca^2+^ currents in hippocampal neurones. Like topiramate, levetiracetam exhibits an excellent pharmacokinetic profile which is featured by rapid absorption through oral administration, linear pharmacokinetics, predominantly renal excretion and insignificant drug interactions^56^. These desirable properties enable levetiracetam to be suitable for treating epilepsy in children who usually require AEDs with high safety and tolerability profiles^57^. As suggested by animal studies, levetiracetam does not exhibit anticonvulsant activity against maximal electroshock seizures, while fully kindled seizures as well as the development of kindling can be effectively attenuated by levetiracetam^57^. Therefore, it may be useful in controlling seizures among patients who are susceptible to posttraumatic epilepsy^58^.

With respect to adverse effects, a study conducted by Neyens *et al*. indicated that levetiracetam as an adjunct AED did not have significant impairment on cognitive functions of chronic epilepsy patients who were treated by standard AEDs^59^. However, the use of levetiracetam may cause several behavioral adverse effects such as hostility and nervousness in children as suggested by previous trials^60^. Although levetiracetam is more tolerable than other AEDs, a cross-sectional study reveals that significantly lower bone mineral density (BMD) was presented in patients who are treated by levetiracetam and thereby suggesting an unfavorable effect of levetiracetam on bone health^61^. Therefore, levetiracetam should be selected with caution especially for infants and children. The recommended starting dose of levetiracetam for adults is 1000 mg/day, while the corresponding starting dose should be reduced to 250 mg at bedtime for those with higher risk of psychiatric adverse effects^60^. As mentioned earlier in our study, identifying factors that can be used to predict response to a specific AED such as levetiracetam can be very challenging and ongoing researches should be devoted to overcoming this challenge.

Our study also provided evidence that lamotrigine was relatively less efficacious with respect to the 50% RR in comparison to other AEDs. Lamotrigine is considered as a good initial monotherapy option for epilepsy patients, while this suggestion is not supported by our analysis potentially due to the fact that only one study comparing pregabalin, lamotrigine and placebo was incorporated in our analysis. Therefore, the lack of evidence and comparisons may conceal the true effectiveness of lamotrigine which has been demonstrated by retrospective studies. For instance, a retrospective study conducted by Arif *et al*. indicates that lamotrigine exhibits the highest retention rate (79%) as well as the highest seizure-free rate (54%) over a period of 12 months^62^. However, this retrospective study was carried out in older adults with epilepsy and hence the corresponding results may not be generalized to other populations. Besides that, the tolerability of lamotrigine with respect to dizziness and somnolence cannot be assessed by our NMA due to the lack of evidence. Thus, extensive systematic review should be conducted in the future in order to ascertain the relative efficacy and tolerability of lamotrigine.

Several limitations of our study should also be acknowledged. Firstly, our analysis is merely based on randomized trials and may produce inconsistent results as compared to those obtained from retrospective studies. Most of included RCTs were placebo-controlled experiment, lead to a lack of evidence for direct comparison between different treatments. The results of comparison between different treatments came from indirect evidence mostly hence it was hard to assess the consistency of the results. Besides, the selection of endpoints with respect to the efficacy and tolerability of AEDs may differ from those of other studies. For instance, one popular approach to evaluate the long term performance of AEDs is to assess their corresponding retention rates which reflect the efficacy, safety as well as the willingness of patients to continue treatment simultaneously. However, we are unable to cope with studies in which the retention rate is considered as the primary endpoint since it is unfeasible to interchange between the retention rate and 50% responder rate. Furthermore, randomized trials included in our study may be carried out in different populations and varied medication compliance or adherence may have significant influence on the long-term efficacy.

## Conclusions

In conclusion, we conducted a methodologically and statistically rigorous analysis of second and third generation AEDs and indicated that topiramate appears to be the most efficacious AED and levetiracetam demonstrates balanced effectiveness and tolerability. Since the main objective of treating epilepsy with AEDs is to control seizure without significant side effects, our review may provide guidance for clinical decision-making and optimizes resource allocation. Also, the valid prediction of responses to AEDs should be proposed as the next step in this research area.

## Electronic supplementary material

Supplementary Information
