# Supplementary material for: Efficacy and Tolerability of Second and Third Generation Anti-epileptic Drugs in Refractory Epilepsy: A Network Meta-Analysis
Source: Sci Rep. 2017 May 31;7:2535. doi: 10.1038/s41598-017-02525-2 (PMC5451432; doi:10.1038/s41598-017-02525-2)

Efficacy and Tolerability of Second and Third Generation Anti-epileptic Drugs in Refractory Epilepsy: A Network Meta-Analysis

Running title: A network meta-analysis of trials about AEDs in refractory epilepsy

Chuanjun Zhuo[1, 2, 3, 4], Ronghuan Jiang[5], Gongying Li[3], Mingjing Shao[6], Ce Chen[1], Guangdong Chen[1], Hongjun Tian[2], Jie Li[2], Rong Xue[7*], Deguo Jiang[1*]

[1]Department of Psychiatry, Wenzhou Seventh People's Hospital, Wenzhou, Zhejiang, 325005, China

[2]Department of Psychiatry, Tianjin Anding Hospital, Tianjin, 300222, China

[3]Institute of Mental Health, Jining Medical University, Jining, Shandong, 272067, China

[4]Department of Psychiatry, Tianjin Anning Hospital, Tianjin, 300222, China

[5]Department of Psychological Medicine, Chinese PLA (People's Liberation Army) General Hospital, Chinese PLA (People's Liberation Army) Medical School, Beijing, 100853, China

[6]Department of Cardiology, China-Japan Friendship Hospital, Beijing, 100029, China

[7]Department of Neurology, Tianjin Medical University General Hospital, Tianjin, 300052, China

Corresponding to: Rong Xue, Department of neurology, Tianjin Medical University General Hospital, No.154 Anshan Road, Peace Street, Tianjin, 300052, China

Email: yyc_dr@yeah.net

Tel/Fax: +86 022-60362255

Corresponding to: Deguo Jiang, Department of psychiatry, Wenzhou Seventh People's Hospital, No. 552 East Xishan Road, Ouhai District, Wenzhou, Zhejiang, 325005, China

Email: fengsqing@126.com

Tel/Fax: +86 0577-88209928/88432238

Chuanjun Zhuo and Ronghuan Jiang contributed equally to this work.

Supplementary information

Figure S1 Details of flow chart.

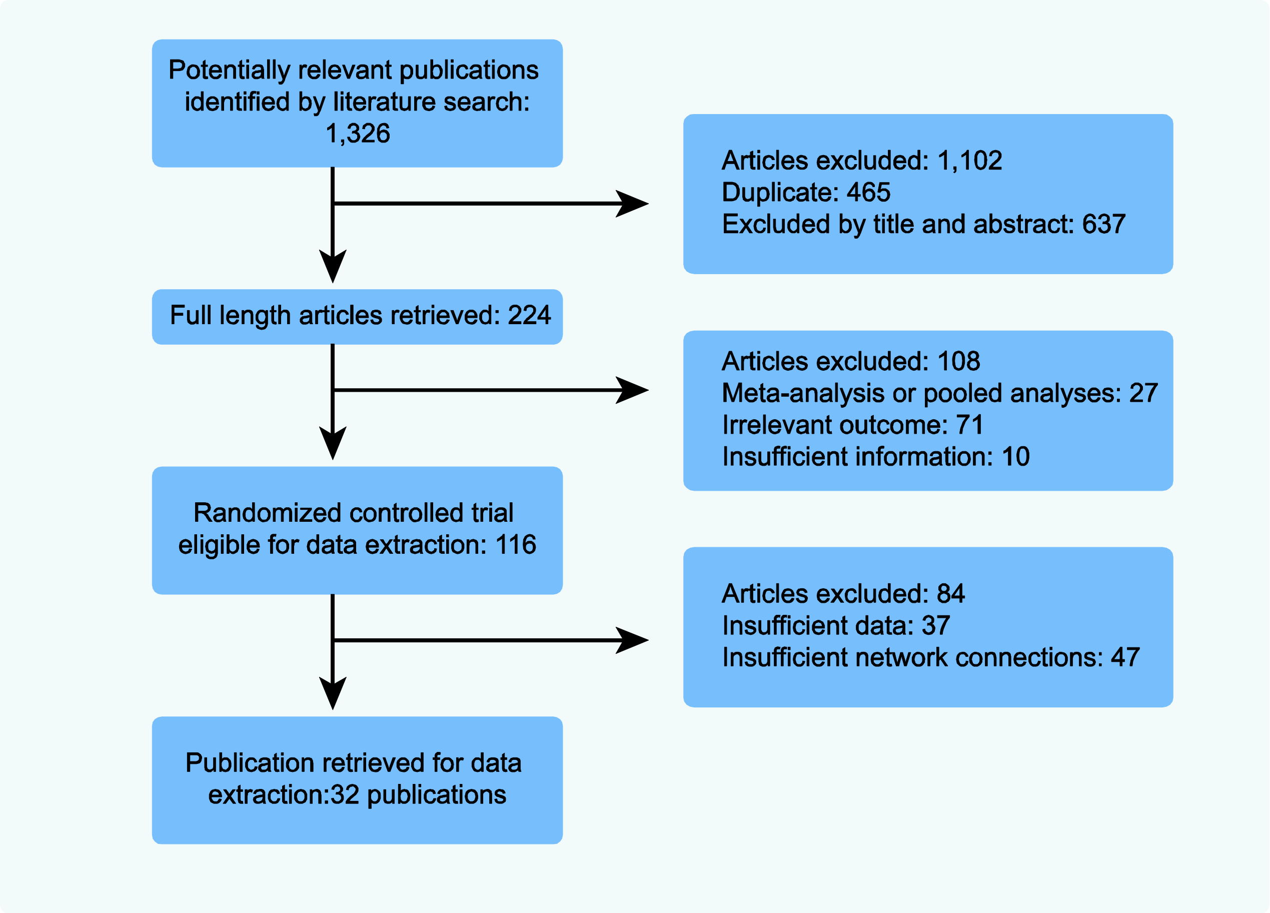

Supplement: Supplementary file 1 — Supplementary Information [file 41598_2017_2525_MOESM1_ESM.pdf]
